# Designed gRNAs for CRISPR-Cas9 based antifungal resistance in eggplant

**DOI:** 10.6026/97320630019844

**Published:** 2023-08-31

**Authors:** Archana Prajapati, Vikrant Nain, Deepali Singh

**Affiliations:** School of Biotechnology, Gautam Buddha University, Greater Noida 201312, India

**Keywords:** CRISPR/Cas9, fungal pathogens, gRNA, Eggplant

## Abstract

Eggplant is an important vegetable crop and is a good source of antioxidants, minerals, and vitamins. It has been used in ancient medicines for the treatment of multiple diseases. However, the cultivated varieties of eggplant are susceptible to numerous pathogens and pests that have a negative impact on vegetable crops. Increased resistance achieved through resistance genes (R genes) is limited in eggplant breeding due to the fact that R genes are typically specific to a pathogen race and can be quickly surpassed by pathogen evolution. The susceptibility genes (S genes) in plants facilitate pathogen entry and proliferation, thus disabling these genes might be beneficial for providing a broad range and durable resistance against pathogens. Reports on crops such as *Arabidopsis*, rice, wheat, citrus, and tomatoes have highlighted that the knockout mutants of the S genes are tolerant to multiple different pathogens. The CRISPR/Cas9 system facilitates plant genome editing that can be utilized efficiently for crop improvement. In the current work, we have identified the homologs of candidate S genes DMR1, DMR6, EDR1, and PMR4/5/6 in the eggplant genome and designed and screened putative gRNAs against the identified target loci. The gRNAs were screened and selected on the basis of recognition of the PAM sequence, the MIT score, their minimum free energy, and the secondary structure. Five gRNAs for each gene homolog were selected after an in-depth analysis of all the predicted gRNAs using the above-mentioned criterion.

## Background:

**Figure 1:**
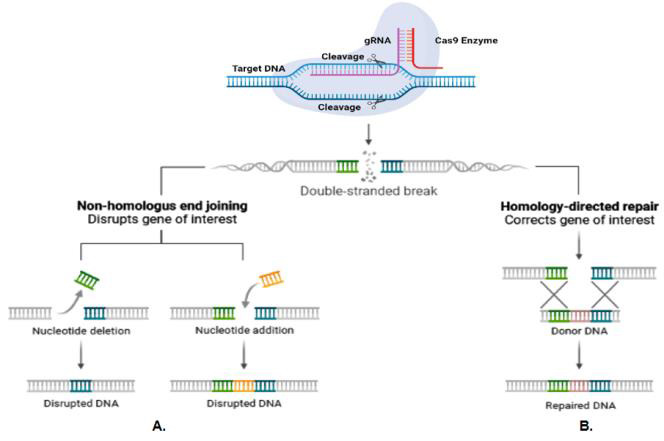
CRISPR/Cas9 Gene Editing System: CRISPR/Cas9 gene editing system requires Cas9 and gRNA. Cas9 endonuclease cleaves target DNA at 3-nt upstream of the PAM motif. Cas9-induced DBSs activate NHEJ or HDR DNA repair. A. NHEJ repairs the DSB without a homologous DNA template, but it is error-prone and causes short insertions or deletions (indels) at the cleavage site. B. HDR repairs the DSB with a homologous DNA template, leading to precise mutations. Created with BioRender.com

The global population is steadily increasing leading to a corresponding increase in food demand. By 2050, there will be approximately 9.7 billion people on the planet, prompting a 70% increase in food production to meet nutritional needs. To ensure food and nutrition security, disease-resistant high-yielding, stress-tolerant, and highly nutritious crop varieties must be developed [1]. *Solanum melongena* L., commonly referred to as eggplant, aubergine, and brinjal, is an agronomically and economically significant non-tuberous *Solanaceae* vegetable. Eggplants have been cultivated in Europe, Asia, Africa, and the Middle East for centuries. It is rich in antioxidants (anthocyanins and phenolic acids), which are advantageous to human health [2,3]. Plant diseases are one of the leading causes of crop yield losses worldwide. Plant diseases caused by obligate biotrophic fungi and oomycetes include powdery mildews, rusts, and downy mildew [4]. Our understanding of plant-pathogen interaction has increased with the advancement of knowledge about microbial components required for plant-pathogen interaction, as well as the cloning of resistance genes (R) involved in race-specific disease resistance. The majority of these genes are membrane receptors that trigger resistance against pathogens by interacting with the pathogen's cognate avirulence gene products. Following a specific interaction, an array of host defense responses are activated leading to successful inhibition of pathogen spread and infection [5]. It has been observed that the mutation of non-essential susceptibility genes (S) can lead to a decrease in pathogen growth and the generation of disease-resistant varieties or mutants [6], [7], [8]. In fact, the loss of function of S genes provides an excellent strategy for durable pathogen resistance for crop improvement. In comparison to R-gene mediated resistance, the S-gene mediated resistance can last longer since the pathogen needs to adapt and interact with the host in the absence of the S-gene products. Currently, there are relatively few examples of S-gene-deficient varieties used in agriculture; nevertheless, the control of S-gene-mediated plant susceptibility represents a promising strategy for reducing disease in crops [9]. In recent years, genetic studies on the model plant *Arabidopsis thaliana* resulted in the identification of a number of genes involved in pathogen susceptibility [4]. *Arabidopsis* ENHANCED DISEASE RESISTANCE1 (EDR1) and POWDERY MILDEW RESISTANT (PMR) genes PMR4/5/6 are susceptibility genes involved in the interaction with powdery mildew fungi. It has been observed that *Arabidopsis* EDR1 mutants are resistant to powdery mildew *Golovinomyces cichoracearum* and the bacterial pathogen *Pseudomonas syringae* [10,11, 12] and the plants with mutations in PMR4/5/6 genes are resistant to powdery mildews [4], [5], [13,14,15]. Downy Mildew Resistance (DMR) gene, DMR1 encodes a homoserine kinase, and its dysfunction causes homoserine accumulation, which is responsible for resistance to downy mildew [10]. Also, inactivation of *Arabidopsis* DMR6 (AtDMR6) tends to raise salicylic acid (SA; 2-hydroxybenzoic acid) levels and confers resistance to a number of pathogens, including bacteria and oomycetes [16], [17]. Recently, genome editing technologies have progressed and become powerful genetic tools for increasing pathogen resistance in plants [18]. These technologies include the use of Zinc-finger nucleases (ZFNs), transcription activator-like effector nucleases (TALENs) or clustered regularly interspaced short palindrome repeats(CRISPR)/CRISPR-associated protein9 (Cas9) [1], [18,19]. The availability of reference genome sequences and the CRISPR/Cas9-editing system has made it possible to develop disease-resistant plants by precise editing of the genes. CRISPR/Cas9 gene editing system requires Cas9 and a single guide RNA (sgRNA), which is a fusion of CRISPR RNA (crRNA) containing a 20-nt DNA target sequence upstream of a Cas9 protospacer adjacent motif (PAM, 5'-NGG-3') and trans-activating CRISPR RNA (tracrRNA) [20], [21]. This technology relies on specific base pairing of the 20-bp sequence of the sgRNA with the target DNA, which directs Cas9 endonuclease to cleave the target DNA at 3-nt upstream of the PAM motif [1], [22]. The double-strand breaks (DSBs) generated by Cas9 activate innate DNA repair by either non-homologous end-joining (NHEJ) or homology-directed repair (HDR) mechanism [23]. Without a homologous DNA template, the cell repairs the DSB through NHEJ, which is error-prone causing short insertions or deletions (indels) around the cleavage site. With a homologous DNA template, the cell will repair the DSB through HDR, leading to the creation of precise mutations (Figure 1). As this approach can generate homozygous or complete knockout mutants as early as in the first generation of transgenic lines for both diploid and polyploid species [20], it greatly speeds up functional genomics studies and shortens the breeding process. CRISPR/Cas9 has been used to silence the sweet basil homolog of *DMR6*, *ObDMR6*, to generate resistance against *P. belbahrii* [22]. *Pmr4* and *dmr6* loss-of function through CRISPR/Cas reduced the susceptibility to PM in tomato plants [24]. In this article, orthologs of S genes AtDMR1, AtDMR6, AtEDR1, AtPMR5, AtPMR6 and SlPMR4 have been identified in eggplant genome. Further, efficient gRNAs were designed and screened to target homologs of these loci in the eggplant genome on the basis of specificity scores, minimum free energies and secondary structure of putative gRNAs etc.

## Materials and Methods:

## Identification of putative homologues of EDR1, DMR1/6, PMR 4/5/6 in eggplant:

Multiple genes with a rather high level of similarity were located in the Eggplant genome. Database SGN (Sol Genomics Network) (https://solgenomics.net/) [25] used and the protein sequence of *Arabidopsis* EDR1, DMR1, DMR6, PMR5 and PMR6 used as a query to identify these homologues in Eggplant genome using Blastp program of SGN [26]. For identification of PMR4 gene in eggplant, tomato Solyc07g053980.2 protein sequence was used as a query.

## gRNA designed against predicted putative homologues of EDR1, DMR1/6, PMR 4/5/6 in eggplant:

CRISPOR online tool used for the identification of potential guide RNAs and potential off-target sites in eggplant genome. CDS sequences of DMR1, DMR6, EDR1, PMR4, PMR5 and PMR6 genes of Eggplant used as query to design gRNA using CRISPOR web based tool (http://crispor.tefor.net/) [27]. In CRISPOR online tool, genome *Solanum melongena*-Eggplant aubergine-Solgenomic.net V3 was selected. 20bp-NGG Protospacer Adjacent Motif (PAM) option was selected to design gRNAs.

## Efficient gRNAs evaluation:

Secondary structures and minimum free energy of top five gRNAs of each gene was calculated using RNAfold web server (http://rna.tbi.univie.ac.at/cgi-bin/RNAWebSuite/RNAfold.cgi) [28].

## Results and Discussion:

Eggplant is one of the highly consumed vegetable crop worldwide and second to tomatoes in terms of nutritional value. However, eggplant is susceptible to a range of fungal, bacterial and insect pests leading to significant crop loss worldwide. CRISPR/Cas9 mediated targeted genome modification has the potential to introduce specific and precise mutation in cultivated genome. With the availability of whole genome sequence, the identification of gene sequences and designing specific changes have become easier. The present study aims at designing and screening of CRISPR/Cas9 guide RNAs against the target susceptibility genes DMR1/6, EDR1, PMR 4/5/6.

Using *Arabidopsis* protein sequence as query, we identified three homologues of DMR1 in eggplant genome: SMEL_009g330290.1.01, SMEL_011g375880.1.01, and SMEL_001g115110.1.01. We obtained two homologues for DMR6: SMEL_003g185630.1.01 and SMEL_006g265100.1.01. Similarly, we identified four homologues of EDR1: SMEL_001g125300.1.01, SMEL_003g195880.1.01, SMEL_006g261980.1.01, and SMEL_008g307780.1.01; two homologues of PMR4: SMEL_000g038330.1.01 and SMEL_007g290290.1.01; one gene for PMR5: SMEL_006g268650.1.01 and two homologues of PMR6: SMEL_000g017800.1.01 and SMEL_011g365710.1.01 [Table 1]. The homologs were located on different chromosomes and were spread across the genome.

**Figure 2:**
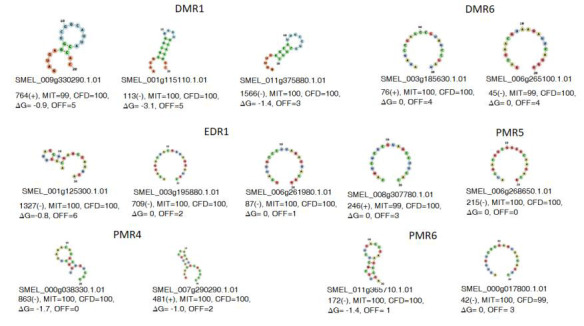
Selection of efficient putative gRNAs against predicted DMR1, DMR6, EDR1, PMR4, PMR5 and PMR5 homologous gene in Eggplant on the basis of MIT and CFD specificity score, least off-target similarity (OFF), minimum free energy (ΔG) and secondary structures of gRNAs.

The CRISPOR and RNA fold online tools were used to design and evaluate guide RNAs against these homologues. For DMR1 homologues SMEL_009g330290.1.01, SMEL_011g375880.1.01, and SMEL_001g115110.1.01, the CRISPOR tool yielded 189, 206, and 243 gRNAs, respectively. We found 104 and 88 gRNAs against DMR6 homologues SMEL_003g185630.1.01 and SMEL_006g265100.1.01, respectively. Similarly, for EDR1 homologues SMEL_001g125300.1.01, SMEL_003g195880.1.01, SMEL_006g261980.1.01, and SMEL_008g307780.1.01, the CRISPOR tool returned 158, 141, 145, and 145 gRNAs, respectively. We obtained 186, 198, and 87 gRNAs against PMR4 homologues SMEL_000g038330.1.01, SMEL_007g290290.1.01, and PMR5 homolog SMEL_006g268650.1.01. We found 158 and 146 gRNAs for PMR6 homologs SMEL_000g017800.1.01 and SMEL_011g365710.1.01, respectively. Next, we selected top three gRNAs of each homolog gene on the basis of MIT and CFD specificity score, least off-target sites in target genome. This resulted in 42 gRNAs (Supplementary Table 1-see PDF). Out of these 42 gRNAs, two gRNAs were selected against each homologue gene on the basis of MIT and CFD specificity score, least off-target similarity, minimum free energy and secondary structure of gRNA (Figure 2). The gRNAs did not show any secondary structure and they all had zero or minimal free energy indicating that they are accessible to binding to the target site with high efficiency.

## Conclusion:

Guide RNAs were designed against the selected S genes for conferring resistance against fungal diseases in eggplant. The predicted gRNAs were screened based on PAM sequence, MIT score and ranking, off target similarity, secondary structure of the gRNAs and their minimum free energy. All these parameters increase the specificity and efficiency of genome editing at the desired loci. The gRNAs can be cloned under a suitable promoter for expression in eggplant using a suitable transformation protocol and the putative transgenics can be screened for the edits in the target genes as well and off targets. The use of CRISPR/Cas9-mediated gene editing allows for the development of foreign DNA-free crops, which is more acceptable by consumers, as opposed to the conventional way of developing genetically modified (GM) crops. The trait generated through CRISPR-mediated gene editing can be segregated from the introduced transgenes; or the desired trait can be achieved via DNA-free approach for delivery of gene-editing reagents. Many proof-of-concept studies have used CRISPR for crop nutritional improvement and enhanced resistance to biotic and abiotic stresses [20]. The resultant transgene-free plants can bypass the regulatory restrictions set for GM crops by U.S. Department of Agriculture.

## List of Abbreviations:

R genes: Resistance genes; S genes: Susceptibility genes; gRNA: guide RNA; PAM: Protospacer Adjacent Motif; EDR: Enhanced Disease Resistance; PMR: Powdery Mildew Resistance; DMR: Downy Mildew Resistance; SA: Salicylic Acid; ZFNs: Zinc-Finger Nucleases; TALENs: Transcription Activator-like Effector Nucleases; CRISPR/Cas9: Clustered Regularly Interspaced Short Palindrome Repeats/CRISPR-associated protein9; sgRNA: single guide RNA; crRNA: CRISPR RNA; tracr RNA: Trans-activating CRISPR RNA; DSBs: Double-strand breaks (DSBs); NHEJ: Non-homologous end-joining; HDR: Homology-directed repair; SGN: Sol Genomics Network; GM: Genetically Modified; ID%: Percent Identity; Aln: Alignment; Chr no.: Chromosome Number

## Figures and Tables

**Table 1:** Eggplant homologues of DMR1, DMR6, EDR1, PMR4, PMR5 and PMR6 along with their chromosomal locations

**DMR1**					
**Gene-ID**	**Chr No.**	**Arabidopsis ortholog**	**ID%**	**Aln**	**E value**
SMEL_009g330290.1.01	9	AT2G17265.1	76.45	250/327	6.00E-163
SMEL_001g115110.1.01	1	AT2G17265.1	76.32	245/321	1.00E-157
SMEL_011g375880.1.01	11	AT2G17265.1	74.46	242/325	7.00E-153
DMR6
Gene-ID	Chr No.	Arabidopsis ortholog	ID%	Aln	E value
SMEL_003g185630.1.01	3	AT5G24530.1	67.06	228/340	0
SMEL_006g265100.1.01	6	AT5G24530.1	67.46	228/338	1.00E-171
EDR1
Gene-ID	Chr No.	Arabidopsis ortholog	ID%	Aln	E value
SMEL_001g125300.1.01	1	AT1G08720.1	54.05	534/988	0
SMEL_003g195880.1.01	3	AT1G08720.1	73.61	198/269	3.00E-143
SMEL_006g261980.1.01	6	AT1G08720.1	74.35	200/269	5.00E-143
SMEL_008g307780.1.01	8	AT1G08720.1	68.97	200/290	7.00E-137
PMR4
Gene-ID	Chr No.	Tomato ortholog	ID%	Aln	E value
SMEL_000g038330.1.01		Solyc07g053980.2	94.17	1665/1768	0
SMEL_007g290290.1.01	7	Solyc07g053980.2	93.59	1665/1779	0
PMR5
Gene-ID	Chr No.	Arabidopsis ortholog	ID%	Aln	E value
SMEL_006g268650.1.01	6	AT5G58600.2	58.36	164/281	8.00E-125
PMR6
Gene-ID	Chr No.	Arabidopsis ortholog	ID%	Aln	E value
SMEL_011g365710.1.01	11	AT3G54920.1	67.62	284/420	0
SMEL_000g017800.1.01		AT3G54920.1	65.95	277/420	0
